# Effects of Label Noise on Deep Learning-Based Skin Cancer Classification

**DOI:** 10.3389/fmed.2020.00177

**Published:** 2020-05-06

**Authors:** Achim Hekler, Jakob N. Kather, Eva Krieghoff-Henning, Jochen S. Utikal, Friedegund Meier, Frank F. Gellrich, Julius Upmeier zu Belzen, Lars French, Justin G. Schlager, Kamran Ghoreschi, Tabea Wilhelm, Heinz Kutzner, Carola Berking, Markus V. Heppt, Sebastian Haferkamp, Wiebke Sondermann, Dirk Schadendorf, Bastian Schilling, Benjamin Izar, Roman Maron, Max Schmitt, Stefan Fröhling, Daniel B. Lipka, Titus J. Brinker

**Affiliations:** ^1^National Center for Tumor Diseases, German Cancer Research Center, Heidelberg, Germany; ^2^Department of Medicine III, RWTH University Hospital Aachen, Aachen, Germany; ^3^Department of Dermatology, Heidelberg University, Mannheim, Germany; ^4^Skin Cancer Unit, German Cancer Research Center, Heidelberg, Germany; ^5^Skin Cancer Center at the University Cancer Centre and National Center for Tumor Diseases Dresden, Dresden, Germany; ^6^Department of Dermatology, University Hospital Carl Gustav Carus, Technische Universität Dresden, Dresden, Germany; ^7^Berlin Institute of Health (BIH), Charité, Berlin, Germany; ^8^Department of Dermatology and Allergology, Ludwig Maximilian University of Munich, Munich, Germany; ^9^Department of Dermatology, Venereology and Allergology, Charité–Universitätsmedizin Berlin, Berlin, Germany; ^10^Dermatopathology Laboratory, Friedrichshafen, Germany; ^11^Department of Dermatology, University Hospital Erlangen, Erlangen, Germany; ^12^Department of Dermatology, University Hospital Regensburg, Regensburg, Germany; ^13^Department of Dermatology, University Hospital Essen, Essen, Germany; ^14^Department of Dermatology, University Hospital Würzburg, Würzburg, Germany; ^15^Department of Medical Oncology, Dana-Farber Cancer Institute, Boston, MA, United States; ^16^Translational Cancer Epigenomics, Division of Translational Medical Oncology, German Cancer Research Center (DKFZ), Heidelberg, Germany; ^17^Faculty of Medicine, Medical Center, Otto-von-Guericke-University, Magdeburg, Germany

**Keywords:** dermatology, artificial intelligence, label noise, skin cancer, melanoma, nevi

## Abstract

Recent studies have shown that deep learning is capable of classifying dermatoscopic images at least as well as dermatologists. However, many studies in skin cancer classification utilize non-biopsy-verified training images. This imperfect ground truth introduces a systematic error, but the effects on classifier performance are currently unknown. Here, we systematically examine the effects of label noise by training and evaluating convolutional neural networks (CNN) with 804 images of melanoma and nevi labeled either by dermatologists or by biopsy. The CNNs are evaluated on a test set of 384 images by means of 4-fold cross validation comparing the outputs with either the corresponding dermatological or the biopsy-verified diagnosis. With identical ground truths of training and test labels, high accuracies with 75.03% (95% CI: 74.39–75.66%) for dermatological and 73.80% (95% CI: 73.10–74.51%) for biopsy-verified labels can be achieved. However, if the CNN is trained and tested with different ground truths, accuracy drops significantly to 64.53% (95% CI: 63.12–65.94%, *p* < 0.01) on a non-biopsy-verified and to 64.24% (95% CI: 62.66–65.83%, *p* < 0.01) on a biopsy-verified test set. In conclusion, deep learning methods for skin cancer classification are highly sensitive to label noise and future work should use biopsy-verified training images to mitigate this problem.

## Introduction

Deep learning (DL) has revolutionized non-medical image analysis and is starting to change clinical workflows. DL can detect cancer in radiological images (1), can predict molecular changes from histology of cancer (2) and can be used to classify dermatoscopic images (3–6). Based on a large amount of input data and the corresponding class labels, the parameters of a neural network are optimized during the training phase in such a way that for an unknown input the predicted output ideally corresponds to the true class label. Both the input and the class labels are generally noisy, whereby the so-called feature noise has less dramatic effects on the classification quality than the label noise (7).

This is particularly the case in medical applications, since the available medical data sets are normally small and cost-intensive knowledge of experts is required for labeling to be as noise free as possible. Furthermore, there are high inter- and intra-rater variabilities in many medical classification tasks, which additionally increase label noise.

For example in dermatology, the visual inspection of a skin lesion show a significant error rate with respect to the gold standard pathology. In the binary classification task melanoma vs. nevi an average sensitivity and specificity of 82 and 59% is reported in Marchetti et al. (8). The same classification task is also investigated in Haenssle et al. (4), where the dermatologists achieved an average sensitivity and specificity of 86.6 and 71.3%, respectively.

In this paper, the effect of label noise on the performance of convolutional neural networks (CNNs) is to be investigated using the binary classification task between melanoma and nevus. In contrast to many existing studies, label noise is not generated artificially by random noise processes (9, 10), but rather by real-world labels that are either from: (a) several dermatologists or (b) the histopathological diagnosis of a biopsy. The latter diagnostic method has a statistically lower error rate and, therefore, most existing work in digital skin diagnosis attaches great importance to a biopsy-verified test set. However, due to the low availability and the high costs of acquiring biopsy-verified images a large amount of non-biopsy-verified images are often included in the training set whose diagnosis are based solely on a consensus decision of several dermatologists or on the lack of temporal lesion changes over several skin examinations (3, 8, 11–13). In doing so, high label noise is introduced in the modeling process. The hypothesis of this paper is that although the CNN learns the diagnostic performance of dermatologists through such a procedure, the performance for skin cancer classification with respect to the gold standard biopsy is severely limited.

This paper utilizes results of a previous reader study (6) where a total of 804 biopsy-verified images (402 melanoma and 402 nevi) were classified by several German dermatologists. For training and evaluating the CNN model two different ground truths are applied. As a first ground truth, the majority decision (MD) of the dermatologists involved in the reader study is considered for each image without taking the biopsy result into account. This means that both biopsy-verified melanoma can be labeled as nevi as well as biopsy-verified nevi can be labeled as melanoma. Furthermore, the result of a histopathological examination of a biopsy (BIO) is used as a second ground truth, which is the gold standard for skin cancer. In order to investigate the influence of label noise on the skin classification task, all combinations of these two different ground truths are applied for training and test set, so that a total of four scenarios can be distinguished: [1] training with MD and testing with MD, [2] training with MD and testing with BIO, [3] training with BIO and testing with MD, and [4] training with BIO and testing with BIO. For each scenario in this setting, we can evaluate the influence of label noise independent of the image selection for training and test set, since for each image both ground truths are available.

## Results

To obtain the labels based on the majority decision of several dermatologists, we sent six electronic questionnaires each containing 134 images of nevi and melanoma to nine German university hospitals. In total, the six questionnaires were completed 144 times (19,296 images were evaluated); 52 questionnaires were filled out by board-certified dermatologists (evaluation of 6,968 images), and 92 by junior dermatologists (evaluation of 12,328 images). Each of the 804 individual images was evaluated by an average of 21.3 dermatologists (median = 21; standard deviation = 4.8; range = 4–31).

The majority decision of dermatologists correctly classified 261 melanomas, 141 melanomas were wrongly classified as nevus. Among the biopsy-verified nevi, 266 were correctly identified by the majority decision, 136 nevi were misclassified as malignant. This results in an overall sensitivity and specificity of 64.9 and 66.2%, respectively. In the subset of 384 images representing the test set, the dermatologists correctly recognize 135 of 188 melanomas, which corresponds to a sensitivity with respect to the gold standard of 71.8%. Considering only the biopsy-verified nevi in the test set, 115 of 196 nevi are correctly classified by dermatologists resulting in an overall specificity of 58.7%.

Training and evaluating a CNN with identical ground truth labels, high accuracies with 75.03% (95% CI: 74.39–75.66%) for dermatological and 73.80% (95% CI: 73.10–74.51%) for biopsy-verified labels can be achieved. However, if the CNN is trained and tested with different ground truths, the accuracy drops significantly. If the CNN is trained with biopsy-verified labels and tested with dermatological labels, accuracy drops significantly to 64.53% (95% CI: 63.12–65.94%, *p* < 0.01). In the contrary case, an accuracy of 64.24% (95% CI: 62.66–65.83%, *p* < 0.01) is achieved on a biopsy-verified test set. Table 1 summarizes the results for the primary and secondary study endpoints for all four scenarios.

**Table 1:** Statistical evaluation of the primary study endpoint (accuracy) and the secondary study endpoints (sensitivity and specificity) for the four different scenarios (training with ground truth majority decision (MD)/testing with ground truth biopsy (BIO), training with MD/testing with BIO, training with BIO/testing with MD, and training with BIO/testing with BIO).

**Ground truth for training**	**MD**		**BIO**	
**Ground truth for testing**	**MD**	**BIO**	**MD**	**BIO**
Mean accuracy	75.03%	64.24%	64.53%	73.80%
95% CI accuracy	74.39–75.66%	62.66–65.83%	63.12–65.94%	73.10–74.51%
Mean sensitivity	76.76%	69.65%	64.31%	75.98%
95% CI sensitivity	75.36–78.15%	67.92–71.37%	62.74–65.88%	74.69–77.26%
Mean specificity	73.00%	59.05%	64.79%	71.85%
95% CI specificity	71.10–74.90%	56.56–61.54%	63.20–66.38%	71.08–72.61%

**Figure 1:**
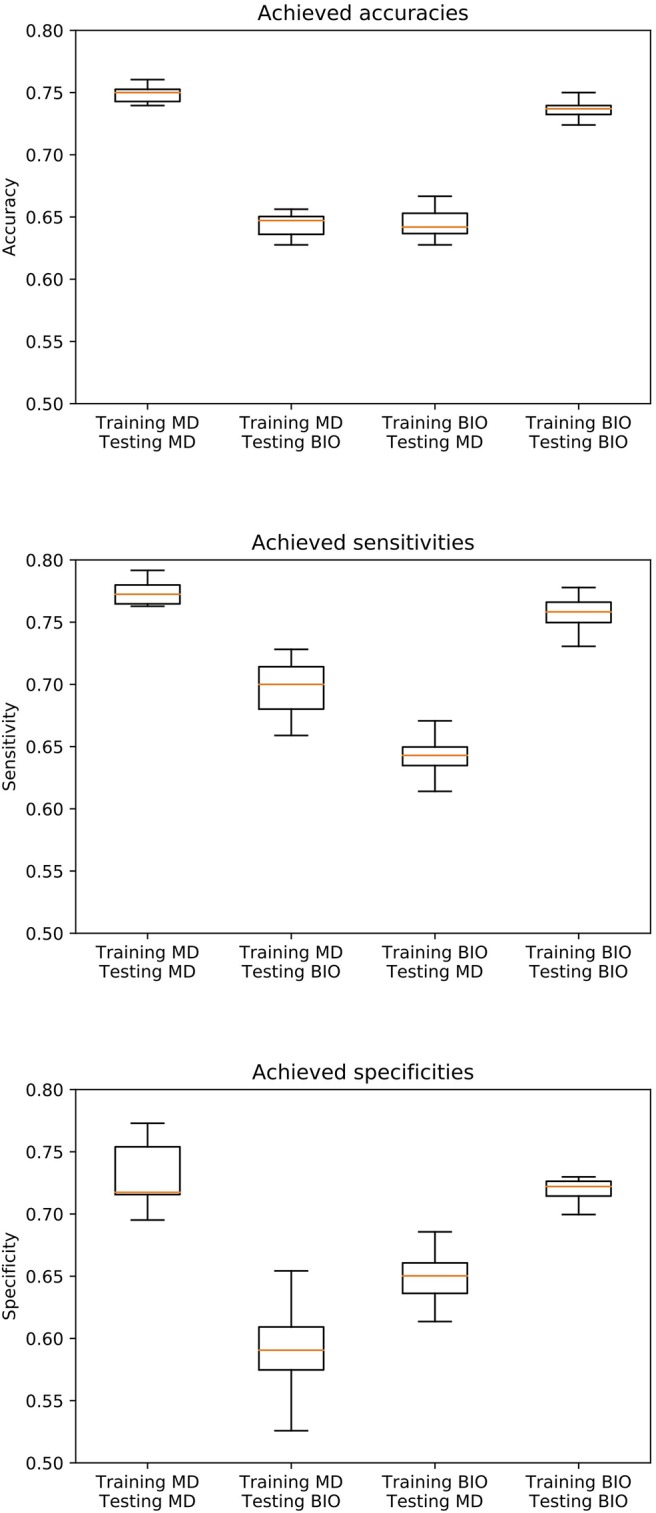
Boxplot of the achieved accuracies, sensitivities, and specificities over 10 simulation runs. 804 biopsy-verified images of nevi and melanoma are labeled by both the majority decision of several dermatologists (MD) and by a biopsy-verified ground truth (BIO). All combinations of these two different ground truths are applied for training and test set, so that a total of four scenarios can be distinguished (MD/MD, BIO/MD, BIO/BIO, MD/BIO). 4-fold cross-validation is applied on a test set of 384 images to evaluate the performance of the algorithm.

**Figure 2:**
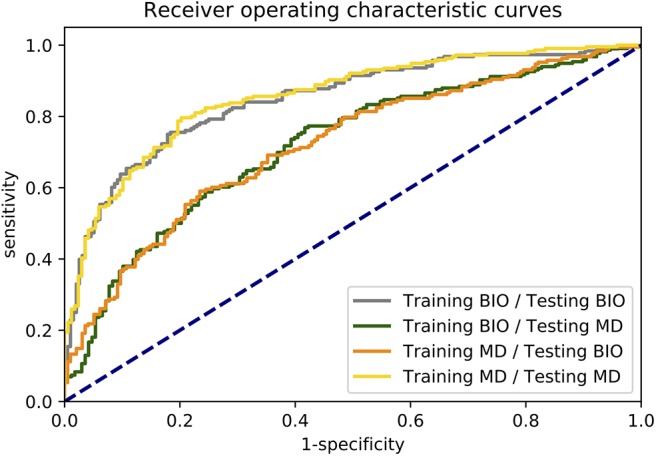
Receiver operating characteristic (ROC) curves of all four scenarios. For calculating the ROC curves, the outputs of each image are averaged over the conducted 10 simulation runs.

In Figure 1, the boxplots for the primary endpoint accuracy as well as the secondary endpoints sensitivity and specificity are depicted for all four scenarios. The receiver operating characteristic (ROC) curves illustrating the relationship between sensitivity and specificity for different cut-off values are shown in Figure 2.

**Figure 3:**
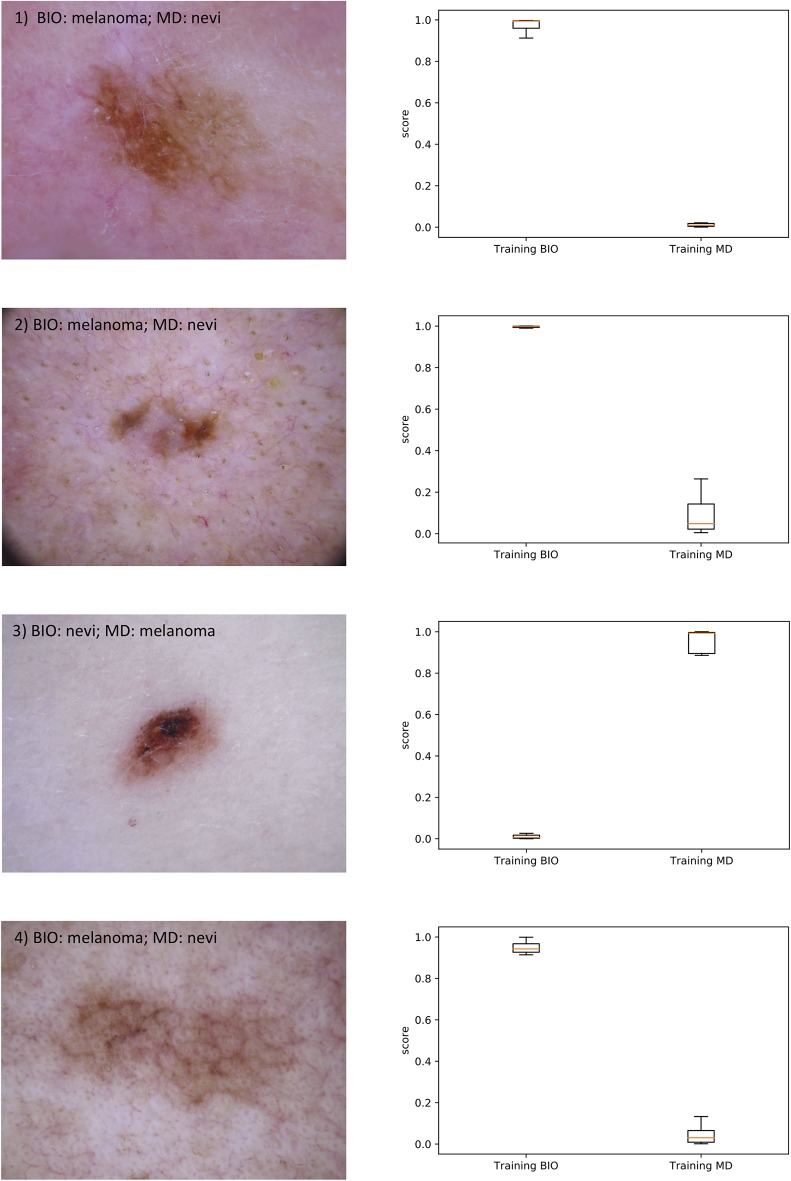
On the left side, the 4 test images are shown for which the outputs of the CNN trained with dermatological labels and those trained with biopsy-verified labels differ the most. In the upper part of each image the majority decision of the dermatologists (MD) and the result of the biopsy (BIO) is given. On the right side, the boxplots of the corresponding outputs of the two CNNs over the 10 conducted simulation runs are presented.

Furthermore, Figure 3 shows on the left side the four test images for which the outputs of the CNN trained with dermatological labels and those trained with biopsy-verified labels differ the most. In the upper part of each image the majority decision of the dermatologists (MD) and the result of the biopsy (BIO) is given. On the right side, the boxplots of the corresponding outputs over the 10 conducted simulation runs are presented.

## Discussion

If a CNN is trained with the diagnosis of several dermatologists, it achieves high-quality results on a test set whose labels have also been created by dermatologists. Convolutional neural networks are therefore able to identify the features in an image that dermatologists are looking for and use them as basis for decision-making. However, with this combination of ground truths it also learns the sources of errors in the dermatological decision. Comparing the achieved sensitivity and specificity with 76.76 and 73.0% with the performance of the dermatologists on the test set (71.8 and 58.7%), the two metrics are substantially higher. This difference may be caused by learning a systematic error of the dermatological diagnosis.

If, however, a CNN trained with majority decisions is tested on a test set with biopsy-verified ground truths, the statistical metrics decrease significantly. In detail, the average accuracy drops from 75.03 to 64.24%. Here it is particularly interesting that the average sensitivity and specificity with 69.65 and 59.05% are in similar orders of magnitude as the statistical metrics of the dermatological diagnoses on the 384 test images regarding the gold standard, i.e., the included.

If a CNN is trained with biopsy-verified images, the accuracy of 73.80% in a test with biopsy-verified images is a little bit lower than the results of a CNN trained and evaluated with dermatological labels. Therefore, learning the relationship between image and corresponding biopsy-verified class labels seems to be a bit harder than learning to mimic the dermatological diagnosis. However, in a test with images whose labels were created by majority decisions of dermatologists, the accuracy also drops to 64.53%. This is also within the range of the label noise.

In all considered scenarios, the accuracies achieved by dermatologists are lower than the reported performance from clinical routine. This is probably due to the selection bias. As all lesions in the study were biopsied, they represent edge cases in general and are naturally difficult to classify. It can be assumed that the accuracy will increase if simpler cases are added to the test set.

There are some limitations of the study. While claiming that deep learning models for skin cancer classification are strongly affected by label noise, only one specific CNN architecture, namely ResNet50, is investigated, thus questioning whether this is truly representative of deep learning models in general. However, the performance of the most common architectures (ResNet, AlexNet, VGGNet, Inception) have been shown to be comparable for most classification tasks.

Furthermore, the overall number of 804 test and training images is very small and as a result the achieved accuracy, sensitivity, and specificity on a biopsy-verified test set are marginally lower than in our previous studies. However, it can be reasonably expected that the relations between the results of the individual scenarios will be comparable for a larger number of images.

## Materials and Methods

The reader study (6) included 804 images with biopsy-verified labels (402 melanoma and 402 nevi), all of which randomly selected from the publicly available database HAM10000 and the ISIC archive (14). These images were sent to dermatologists from nine German university hospitals via six randomly assigned electronic questionnaires, each containing 134 images of either melanoma or nevi. In addition to the classification, the dermatologists were also asked to assess the quality of the images. Only images with at least “sufficient” image quality (“excellent,” “good,” “sufficient”), as rated by the participating dermatologists were included in this study.

To train a CNN with the small number of 804 available images we apply cross-validation which partitions a sample of data into complementary subsets. Since some of the databases in the ISIC archive contain multiple images of the same lesion and the identification of these duplicates is not generally reliably possible with the metadata provided, we restrict the test set to images of HAM10000, where duplicates can be identified by the provided attribute “lesion_id.” This results in 384 images remaining for the evaluation of the algorithm, which are splitted into 4 folds for cross-validation. When testing with one of these splits, all images from the ISIC archive as well as all images with different lesions IDs from HAM10000 from the reader study are used for training.

We conducted 10 simulation runs with a pre-defined training procedure and fixed hyperparameter, in each of which a random process generates a mutually disjoint training and test set according to the above-mentioned rules.

### Training of the CNNs

A ResNet-50 pre-trained on the ImageNet database was selected for the classification of melanoma and nevi. A complete training run consisted of 14 epochs.

During training, the network was first trained in a frozen state, where only fully-connected layers were trained at a high learning rate. This was followed by a step where the complete network was trained using differential learning rates. Earlier layers, which were already pre-trained and fine-tuned to detect general features, such as basic shapes and color gradients, were trained on low values. The further the layer is from the input, the larger the learning rate becomes until a pre-set maximum is reached. Differential learning rates allow for a stronger modification of the later layers, which represent application-specific features, and therefore need more adjustment than pre-trained earlier layers.

All work was carried out in Python 3.7.3 and fastai 1.0 for model.

### Statistical Evaluation

The results of the 10 simulation runs are statistically evaluated by the python modules scikit-learn (version 0.21) and scipy (version 1.3). Throughout the paper, the cut-off value is set to 0.5, i.e., if the output of the CNN is higher than 0.5 or equal, the corresponding input image is classified as melanoma, otherwise as nevi. Since the test set is nearly balanced with respect to the two considered classes, accuracy as a scalar measure of classification quality is a suitable choice and therefore represents the primary endpoint of this study. In order to evaluate the influence of the label noise as a function of the two considered classes, sensitivity and specificity are additionally calculated as the secondary study endpoints. The primary and secondary endpoints are reported by means and confidence intervals calculated based on the corresponding metrics over the four splits of cross validation. In order to illustrate the relationship between sensitivity and specificity the receiver operating characteristic (ROC) curve is calculated for each CNN based on the averaged outputs of each image over the conducted 10 simulation runs. For statistical testing, we use the Wilcoxon test, which is a non-parametric statistical hypothesis test and compares two related samples. Throughout the paper, the significance level is set to 0.05.

## Data Availability Statement

All training and test images used in this study come from the publicly available ISIC archive (www.isic-archive.com).

## Ethics Statement

Ethics approval for the online survey was waived by the ethics committee of the University of Heidelberg due to the fact that all of the participants remained anonymous. Ethical approval for this study was not required in accordance with local legislation and national guidelines.

## Author Contributions

AH, JK, EK-H, RM, MS, and TB contributed to the study concept and design. AH carried out the study implementation, data collection and statistical analysis with contributions from RM, EK-H, and JU. JSU, CB, SH, DS, and BS co-organized the reader study and have therefore contributed relevant data to this study. FM, FG, JU, LF, JS, KG, TW, HK, CB, MH, WS, BI, SF, and DL provided data analysis and/or interpretation of data for this study. TB oversaw the study, critically reviewed and edited the manuscript and gave final approval. All authors provided critical review and commentary on the draft manuscript and approved the final version. All authors guarantee the integrity and accuracy of this study.

## Conflict of Interest

TB reports owning a company that develops mobile apps (Smart Health Heidelberg GmbH, Handschuhsheimer Landstr. 9/1, 69120 Heidelberg). SH reports advisory roles for or has received honoraria from Pierre Fabre Pharmaceuticals, Novartis, Roche, BMS, Amgen and MSD outside the submitted work. Axel H. reports clinical trial support, speaker's honoraria, or consultancy fees from the following companies: Amgen, BMS, Merck Serono, MSD, Novartis, Oncosec, Philogen, Pierre Fabre, Provectus, Regeneron, Roche, OncoSec, Sanofi-Genzyme, and Sun Pharma, outside, the submitted work. BS reports advisory roles for or has received honoraria from Pierre Fabre Pharmaceuticals, Incyte, Novartis, Roche, BMS and MSD, research funding from BMS, Pierre Fabre Pharmaceuticals and MSD, and travel support from Novartis, Roche, BMS, Pierre Fabre Pharmaceuticals and Amgen; outside the submitted work. JSU is on the advisory board or has received honoraria and travel support from Amgen, Bristol Myers Squibb, GSK, LeoPharma, Merck Sharp and Dohme, Novartis, Pierre Fabre, Roche, outside the submitted work. WS received travel expenses for attending meetings and/or (speaker) honoraria from Abbvie, Almirall, Bristol-Myers Squibb, Celgene, Janssen, LEO Pharma, Lilly, MSD, Novartis, Pfizer, Roche, Sanofi Genzyme and UCB outside the submitted work. FM has received travel support or/and speaker's fees or/and advisor's honoraria by Novartis, Roche, BMS, MSD and Pierre Fabre and research funding from Novartis and Roche. The remaining authors declare that the research was conducted in the absence of any commercial or financial relationships that could be construed as a potential conflict of interest.
